# Biomechanical Comparisons between One- and Two-Compartment Devices for Reconstructing Vertebrae by Kyphoplasty

**DOI:** 10.3390/bioengineering11080795

**Published:** 2024-08-05

**Authors:** Oliver Riesenbeck, Niklas Czarnowski, Michael Johannes Raschke, Simon Oeckenpöhler, René Hartensuer

**Affiliations:** 1 Department of Trauma, Hand and Reconstructive Surgery, University Hospital Münster, Albert-Schweitzer-Campus 1, Building W1, Waldeyerstraße 1, 48149 Münster, Germany; 2 Center for Orthopaedic, Traumatology, Handsurgery and Sports Medicine, Klinikum Aschaffenburg-Alzenau, 63739 Aschaffenburg, Germany

**Keywords:** spinal biomechanics, spinal fracture, vertebral body, kyphoplasty, cement volume, load bearing, lumbar spine, cyclic loading

## Abstract

Background: This biomechanical in vitro study compared two kyphoplasty devices for the extent of height reconstruction, load-bearing capacity, cement volume, and adjacent fracture under cyclic loading. Methods: Multisegmental (T11–L3) specimens were mounted into a testing machine and subjected to compression, creating an incomplete burst fracture of L1. Kyphoplasty was performed using a one- or two-compartment device. Then, the testing machine was used for a cyclic loading test of load-bearing capacity to compare the two groups for the amount of applied load until failure and subsequent adjacent fracture. Results: Vertebral body height reconstruction was effective for both groups but not statistically significantly different. After cyclic loading, refracture of vertebrae that had undergone kyphoplasty was not observed in any specimen, but fractures were observed in adjacent vertebrae. The differences between the numbers of cycles and of loads were not statistically significant. An increase in cement volume was strongly correlated with increased risks of adjacent fractures. Conclusion: The two-compartment device was not substantially superior to the one-compartment device. The use of higher cement volume correlated with the occurrence of adjacent fractures.

## 1. Introduction

Balloon kyphoplasty can result in reconstructing vertebral body height in the anterior middle region of vertebrae and in stabilizing vertebrae against compression load [1]. As well as simply reconstructing vertebral body height, restoring the endplate, which is often compressed, seems to prevent posttraumatic disc degeneration in traumatic vertebral body fractures [2]. Kyphoplasty was initially used to treat simple compression fractures, but given its clinical success, its use was extended to treat incomplete burst fractures [3].

In conventional biportal kyphoplasty, a catheter containing one compartment containing one balloon is inserted into each pedicle for a total of two balloons. After a balloon is inflated to reconstruct the vertebra, the balloon is deflated and removed, and the remaining cavity is filled with cement, typically polymethylmethacrylate. Kyphoplasty has undergone refinements ever since an inflatable balloon tamp required for its use received US Food and Drug Administration marketing clearance during 1998 [4].

For achieving the goal of preventing posttraumatic disc degeneration in traumatic vertebral body fractures, different tools for intravertebral reconstruction, all of which are refinements of kyphoplasty, have been developed [5,6,7,8,9,10,11,12,13,14].

However, anatomical endplate reconstruction with elevation remains difficult [15]. Some studies report on specific directional instruments or devices to directly manipulate the endplate but fail to detect a significant benefit considering vertebral body restoration [12,13,14]. A recent development in kyphoplasty potentially addresses this shortcoming by using a device that has a catheter with two compartments, each of which has a balloon [16]. A catheter is inserted into each pedicle for a total of four balloons. Each balloon could be separately filled with contrast medium, making it able to separately elevate the endplate anteriorly and posteriorly.

Considering that incomplete burst fractures vary tremendously and that fracture morphology is crucial to clinical decision-making, we developed a technique to reliably and reproducibly inflict incomplete burst fractures in cadaveric spinal specimens [17,18]. The technique consists of mounting and embedding a specimen into a custom-built frame, inserting the frame into a servo-hydraulic testing machine (Instron 8874, Instron Structural Testing Systems GmbH, Darmstadt, Germany), and applying compression. In all specimens, superior incomplete burst fractures (AO spine classification type A3) were identified [19].

This biomechanical study used this approach to compare the one-compartment catheter (two balloons) device and the two-compartment catheter (two balloons in each compartment) device for the extent of vertebral height reconstruction and load-bearing capacity. The hypothesis was that the two-compartment device was superior to the one-compartment device in both respects. Also, this study sought to evaluate the volume of cement in relation to the risk of subsequent adjacent fractures, a complication of kyphoplasty.

## 2. Materials and Methods

### 2.1. Cadaveric Specimens

In this study, 13 fresh, frozen, female lumbar spine specimens (T11–L3) were used. Specimens were thawed slowly to room temperature and kept moist throughout. All soft tissue and muscles were carefully dissected while preserving the osseous and ligamentous structures in accordance with the recommendations of Wilke et al. [20]. Vertebra L1, which was to be subject to fracture and testing, underwent measurement of the bone mineral density by quantitative computed tomography, as described [18]. Bone mineral densities were categorized according to the 2008 American College of Radiology practice parameter, which was the same as in the 2023 revision, as follows: less than 80 mg/cm^3^, osteoporosis; from 80 mg/cm^3^ to 120 mg/cm^3^, osteopenia; and above 120 mg/cm^3^, normal [21].

### 2.2. Mounting and Embedding Specimens

**Figure 1:**
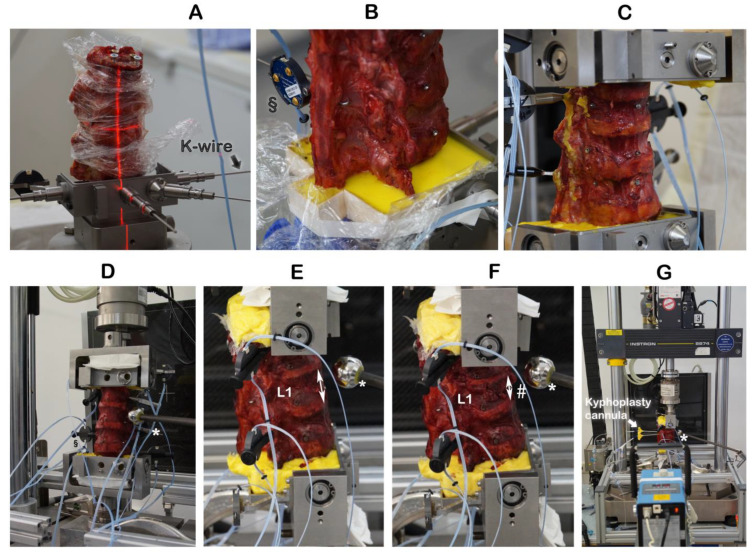
Embedding the specimens and creating incomplete burst fractures. § Motion trackers were not used in this work. ↕ The height of the intact vertebra L1 was measured by a ruler. * Radiographic reference ball; # incomplete burst fracture in L1.

The specimen’s cranial (T11) vertebra and caudal (L3) vertebrae were mounted into the top and bottom, respectively, of a custom-built mounting and embedding frame (Figure 1A–C), where the specimen remained fixed in place for the entire experiment. As described previously, an osteotomy-like procedure to weaken the cranial endplate of the target vertebra, L1, and thus, assure its facture subsequently during compression fracture creation, was performed by using a surgical chisel [18]. To prevent cement in the vertebrae from oozing out through the vertebral lesions resulting from chiseling and so to enable the correct recording of the cement volume, the lesions were sealed on the outside with plastic modeling material (Play-Doh, Hasbro, Pawtucket, RI, USA). The use of this modeling material did not have any effect on stabilizing the vertebrae.

### 2.3. Creating Incomplete Burst Fractures

The cranial endplate of L1 was oriented by laser into a custom-built embedding and mounting frame and held in place by K-wires (Figure 1A,B). The cranial (T11) and caudal vertebrae were embedded with polymethylmethacrylate (Technovit^®^ 3040, Kulzer GmbH, Hanau, Germany) cement (Figure 1C). With the specimen having been mounted and embedded into the frame and the K-wires removed, the frame was inserted upright into a servo-hydraulic testing machine (Instron 8874, Instron Structural Testing Systems GmbH, Darmstadt, Germany) (Figure 1D). The height of the intact vertebra L1 was measured by a ruler (Figure 1E). The combination of osteotomy-like lesions of the target vertebra L1 and of compression at a speed of 300 mm/s to a defined distance of 67% of the intact L1 height resulted in reproducibly creating an incomplete burst fracture of L1 (Figure 1F), classified as A3 according to the AO spine classification [19].

### 2.4. Kyphoplasty

**Figure 2:**
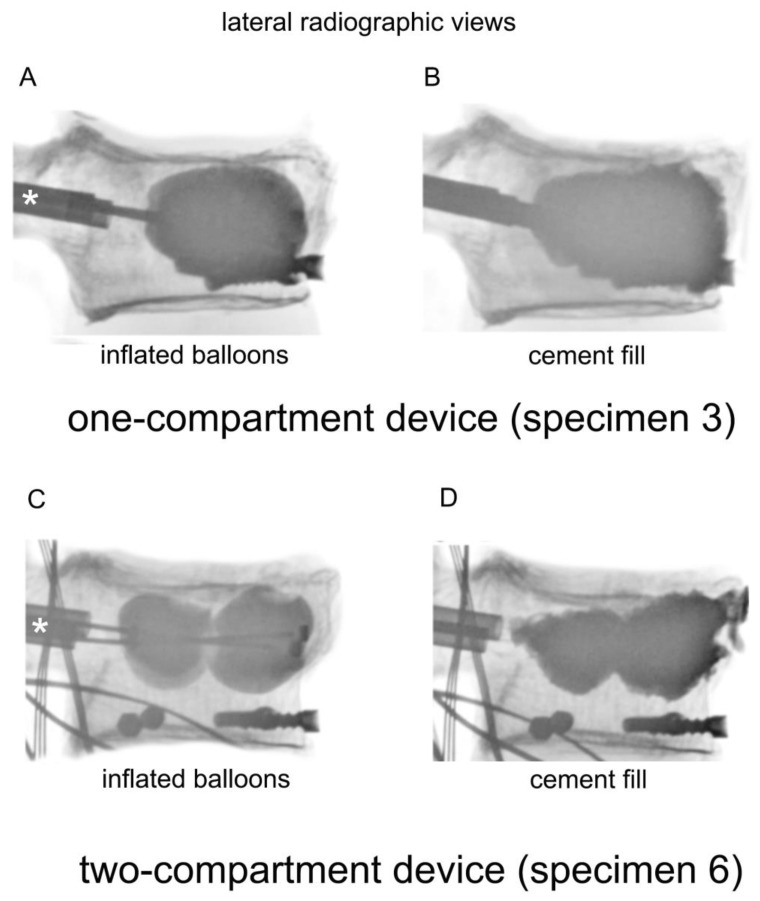
Lateral radiographic views of a one-compartment device with (**A**) inflated balloons and (**B**) cement fill, and a two-compartment device with (**C**) inflated balloons and (**D**) cement fill. The catheters were inserted through the cannulas, which are indicated by asterisks (*).

Kyphoplasty was always carried out according to the manufacturer’s recommendations by the same experienced spine surgeon (RH) in a way that simulates actual surgery. Kyphoplasty cannulas were inserted into vertebra L1 (Figure 1G). Under radiographic guidance (Portable X-Ray Unit AJEX 9020H, JPI Healthcare Solutions, Ronkonkoma, NY, USA), balloons were inflated (Figure 2A,C).

**Figure 3:**
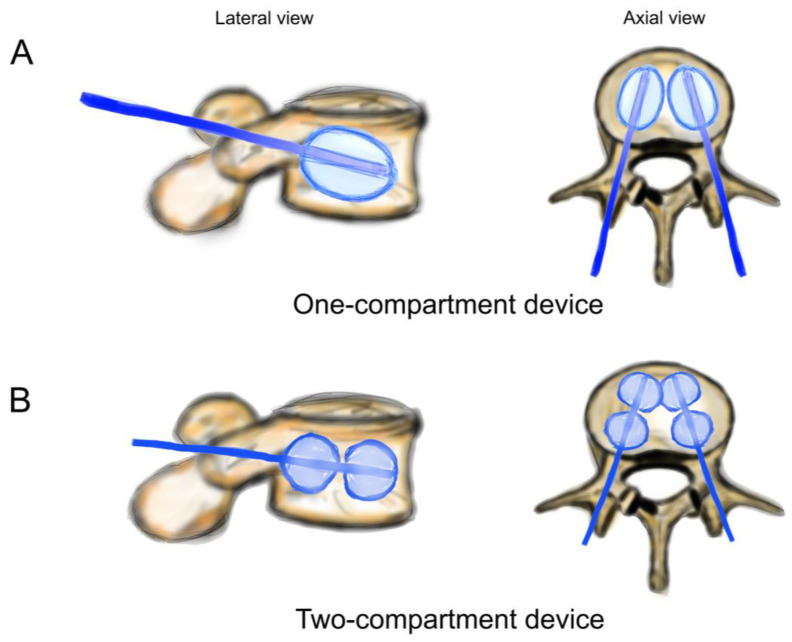
Schematic drawing of catheters and balloons. (**A**) One-compartment device (Joline S9403); (**B**) two-compartment device (Joline S9420).

Both balloons in the one-compartment were inflated, and all four balloons in the two-compartment device were inflated (Figure 3). The one-compartment device (Joline S9403) contained one balloon of 16 mm diameter and 22 mm length with a volume of 6 mL and a maximum pressure of 27 bar. The two-compartment device (Joline S9420) contained two balloons of 16 mm diameter and 8 mm length each, with an individual volume of 3 mL and a maximum pressure of 27 bar.

Reconstruction of the vertebra was monitored by calibrated radiographic examinations according to clinical practice. The extent of inflation depended on surgical judgment. Once the vertebral height was determined to be sufficient, inflation was discontinued, and pressure applied to the balloons was recorded. Then, the balloons were deflated and removed, fully releasing compressive pressure in the apparatus. Having had vertebra T11 fixed into position on the servo-hydraulic machine avoided the collapse of vertebra L1 after the balloons were removed. Polymethylmethacrylate was inserted into vertebrae L1 according to the manufacturer’s recommendations (Figure 2, lower row). The volume of injected polymethylmethacrylate was determined by lateral radiography to simulate clinical practice. The applicated volume was measured using a scale of 0.5 mL increments on the filling device. Vertebral height reconstruction was deemed effective if the surgeon’s impression of height elevation was as observed in actual surgery.

In our model, kyphoplasty was carried out with a specimen mounted upright. Clinically, however, kyphoplasty is undertaken with the patient placed in the prone position. In this position, an axial load acts on the spine from intraabdominal and muscle tension and pressure. Therefore, this pressure needed to be accounted for in our testing. The in vivo studies by Sato et al. and by Wilke et al. published intradiscal pressure in a prone position of 91 kPa and of 0.1 MPa [22,23]. Sato et al. calculated an average spinal load of 144 N. Belkoff et al. calculated a range of 108–212 N from the original data and tested a low axial load (111 N) versus a high axial load (222 N) kyphoplasty scenario, resulting in no significant difference attributed to the axial load [24]. The low load scenario has been used several times in biomechanical testing of kyphoplasty (e.g., [25,26]). In our testing setup, the upper part of the embedding and mounting frame weighs 1.12 kg, which results in an axial compression force of 11 N by gravity. To match the published conditions of 111 N, the servo-hydraulic machine was set to maintain a minimum pressure of 100 N through axial compression on the specimen after fracture and before kyphoplasty.

During balloon inflation in kyphoplasty, the specimen’s height increased, and the pressure started increasing. The pressure was regulated to maintain a constant pressure of 100 N during balloon inflation. When the balloons were deflated, as determined radiographically, and removed, the position of the testing machine was fixed to stop any additional compression, and pressure applied to the balloons was recorded, as already mentioned.

### 2.5. Radiographic Assessment of Vertebral Height Reconstruction

**Figure 4:**
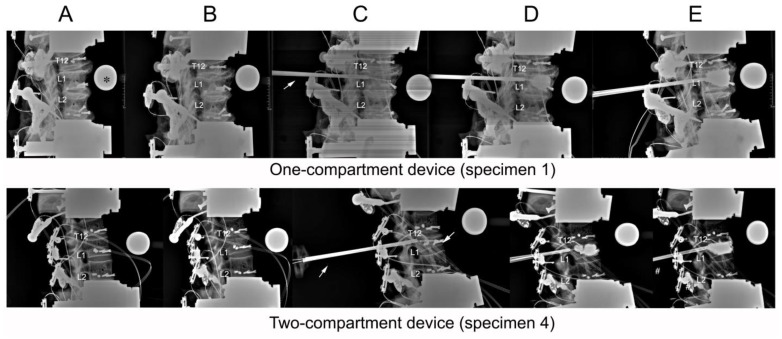
Lateral radiographs taken of (**A**) initial specimens before compression; (**B**) after compression; (**C**) after catheter insertion; (**D**) after balloon inflation; (**E**) and after kyphoplasty. The asterisk (*) in frame (**A**) of the one-compartment group refers to the radiographic reference ball. An arrow in both groups (**C**) points to the cannula.

**Figure 5:**
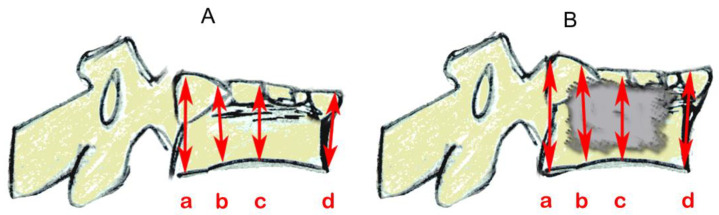
Schematics based on lateral vertebral radiographic projections showing vertebral height measurements (mm) by referring to the referencing ball, as shown in Figure 4. (**A**) after compression and fracture and before catheter insertion and (**B**) after cement insertion. Three of the four measurements were taken in places as per McKiernan et al. [28]: (a) posterior; (b) one-third distance from posterior to anterior; and (d) anterior. The additional location (c), centered between anterior and posterior, was added here.

Radiographs were taken before and after a compression fracture and after kyphoplasty (Figure 4). Measurements of L1 vertebral height were taken at four places (Figure 5) using ImageJ (version 1.53s), an electronic distance measurement tool modified from McKiernan et al. [27,28].

### 2.6. Cyclic Loading Test of Load-Bearing Capacity

**Figure 6:**
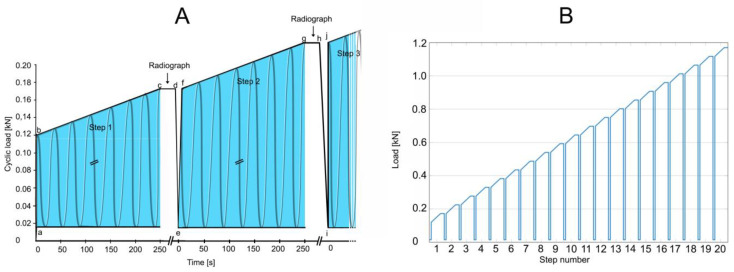
Test of load-bearing capacity by cyclic loading in a stepwise cyclic compression protocol. (**A**) At the beginning of a step (a; e; i), a baseline load of 0.015 kN was applied. Then, through 1.5 s, the load was applied to reach the minimum load for that step (b; f; j). Cyclic loading for that step was applied (b to c; f to g). Each complete 250 s step (the blue areas) consisted of 500 cycles (2 Hz frequency) for 52.5 N per step (slope, 0.21 N/s). With cyclic loading load of a step (c; g), the load was held constant (c to d; g to h) for the taking of a radiograph to examine for failure. When failure occurred, loading was discontinued, and cumulative load was recorded at the moment of failure. If fracture or compression was not observed, then cyclic loading was resumed by reducing to the minimum force of 0.015 kN (d to e; h to i), and a new step began. Cyclic loading was resumed (f; j) by applying the maximum force that had been reached in the previous step (c; g), and the next step began if necessary. (**B**) The test, as depicted in A, was designed for 20 steps. In the event that failure had not occurred by the end of 20 steps, testing was continued by adding additional steps by increasing cyclic loading to 70 N per step for an increased slope of 0.28 N/s.

**Figure 7:**
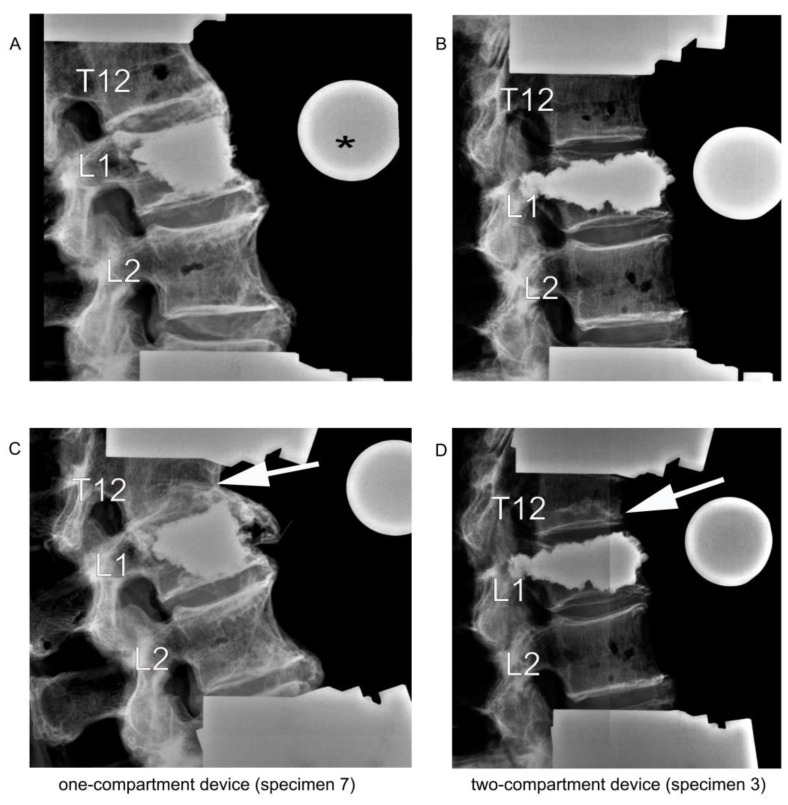
Lateral radiographic images upon kyphoplasty of L1 (**A**,**B**) and after adjacent fracture of T12 (**C**,**D**) resulting from the cyclic loading test of load-bearing capacity. The asterisk (*) in (**A**) refers to the radiographic reference ball. The arrows in (**C**,**D**) point to the fractured T12.

As a test of load-bearing capacity, cyclic loading by a stepwise cyclic compression protocol (Figure 6) was carried out using a servo-hydraulic testing machine and load until failure was recorded. Failure was defined by the radiological appearance of fractures adjacent to vertebra L1 (Figure 7) and/or by a compression depth of 7 cm underneath the starting position. All radiographic images were also examined retrospectively by R. H. to detect any earlier evidence of an adjacent fracture.

Results from the cyclic loading tests were used for two purposes: (1) to compare the two groups for the amount of applied load until failure, and (2) to measure the amount of load applied until failure in relation to the volume of cement that had been used during kyphoplasty.

### 2.7. Statistics

The Mann–Whitney U test was used for group comparisons with a significance level of 0.05. To investigate the relationship between the volume of cement and the appearance of an adjacent fracture, a Spearman correlation analysis with a significance level of 0.025 was used to take into account the effects of multiple testing. Statistical analyses were carried out using SPSS (SPSS^®^ Statistics 27; IBM, Armonk, NY, USA).

## 3. Results

### 3.1. Specimens

Of the 13 specimens, seven were allocated to the one-compartment group and six to the two-compartment group. The median (range) age in years for the one-compartment group was 79 (63 to 94), and for the two-compartment group, 82.5 (67 to 94). The median (range) bone mineral density for the one-compartment group was 71.2 (52.5 to 105.0) g/cm^3^, and for the two-compartment group, 84.8 (68.6 to 120.8) g/cm^3^, which was not a statistically significant difference (*p* = 0.445) All samples except for one in two-compartment group, which had a bone mineral density of 120.8 g/cm^3^, were osteopenic or osteoporotic.

### 3.2. Decreased Vertebral Height after Compression

**Figure 8:**
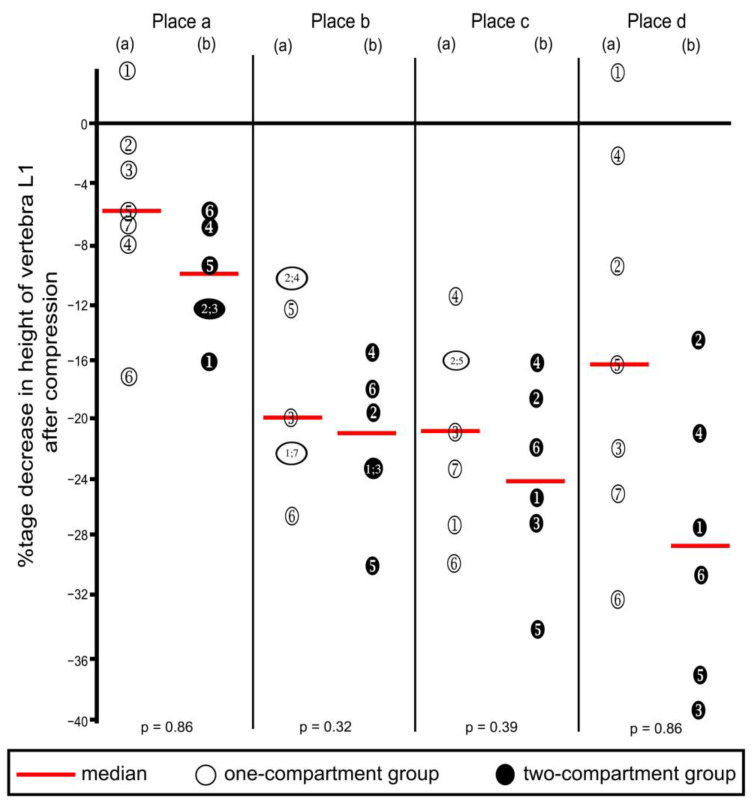
Percentage decreases in vertebra L1 height after compression calculated from the values shown in Table 1. Heights were measured at the four places shown in Figure 5. (a) One-compartment group; (b) two-compartment group. Numbers refer to specimens; *p*-values were calculated by using the Mann–Whitney U-Test.

Compression resulted in decreasing heights of all four places in vertebra L1 (Table 1 and corresponding percentages in Figure 8), except that one increase each was noted in Place A and Place D, in the one-compartment group, as shown in the figure. These increases probably resulted from measuring error, and we inferred that compression did not occur at these two places. Decreases in height were not statistically significantly different between the two groups at any of the four places.

### 3.3. Balloon-Filling and Cement Volumes

The combined median (range) balloon-filling volumes of the two balloons in the one-compartment group was 9.0 (7.0 to 10.0), and of the four balloons in the two-compartment group was 12.5 (11.0 to 14.5) ml. The difference between the two groups was statistically significant (*p* = 0.001). After deflation and balloon removal, the cavity remaining in the vertebra was filled with cement. The median (range) of bipedicular applied cement volume of the one-compartment group was 9.0 (9.0 to 12.0) mL. The median (range) of bipedicular applied cement volume of the two-compartment group was 11.3 (9.0 to 13.5 mL). The difference between the two groups was not statistically significant (*p* = 0.138).

**Figure 9:**
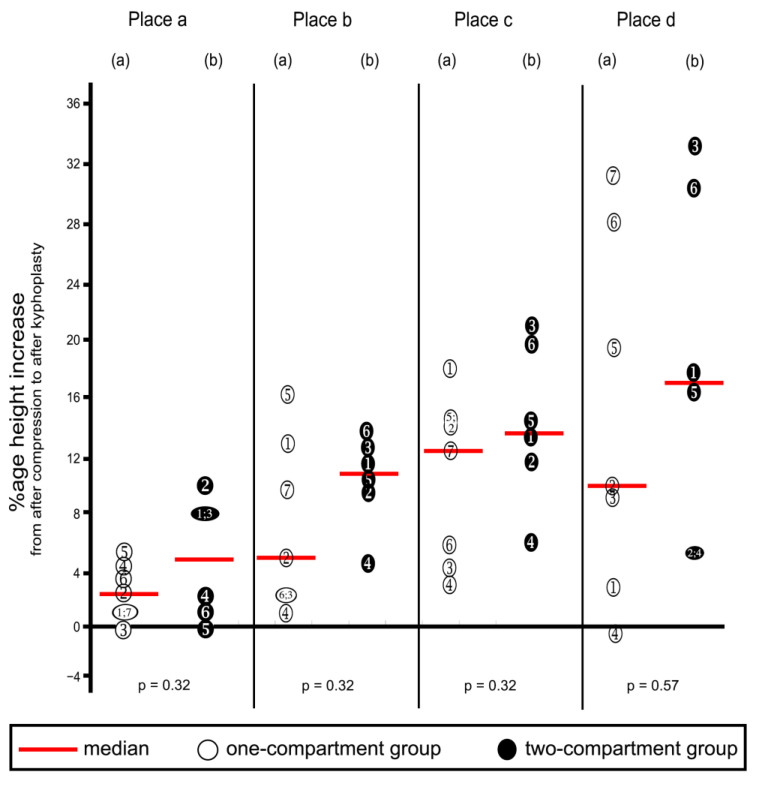
Percentage increases in vertebra L1 height from after compression to after kyphoplasty. Heights were measured at the four places shown in Figure 5. (a) One-compartment group; (b) two-compartment group. Numbers refer to specimens; *p*-values were calculated by using the Mann–Whitney U-Test.

Kyphoplasty resulted in increasing height (endplate elevation) in all four places in vertebra L1 (Table 2 and corresponding percentages in Figure 9), except that decreases were noted in three instances, as shown in the figure. These decreases probably resulted from measuring error. Increases in height were not/ statistically significantly different between the two groups at any of the four places. The surgeon deemed vertebral height reconstruction effective for all specimens.

### 3.4. Cyclic Loading

**Figure 10:**
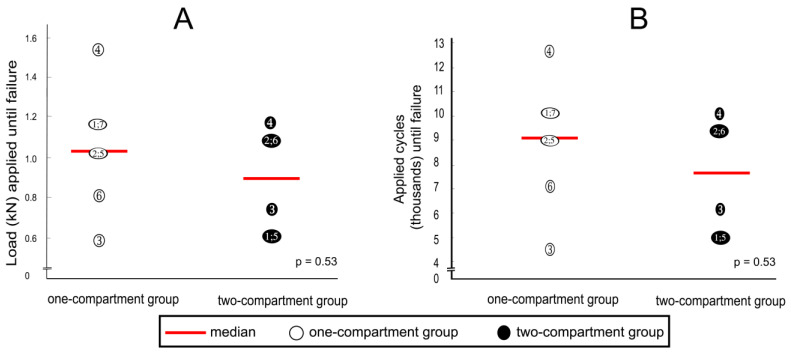
(**A**) applied loads and (**B**) numbers of applied cycles until failure (defined by the radiological appearance of fractures adjacent to vertebra L1 (Figure 7) and/or by a compression depth of 7 cm underneath the starting position. Numbers refer to specimens; *p*-values were calculated by using the Mann–Whitney U-Test.

After cyclic loading, refracture of vertebrae that had undergone kyphoplasty was not observed in any specimen, but fractures were observed in adjacent vertebrae. Although the median load to cause fracture of adjacent vertebrae was about 20% less in the two-compartment group than in the one-compartment group, this difference was not statistically significantly different (Figure 10A). A similar difference could be observed in the number of applied cycles that resulted in failure. Although the median number of applied cycles to result in failure was about 15% less in the two-compartment group than in the one-compartment group, this difference was also not significantly different statistically (Figure 10B).

### 3.5. Correlations between Applied Load and Applied Cycles at Time of Failure with Cement Volume

**Figure 11:**
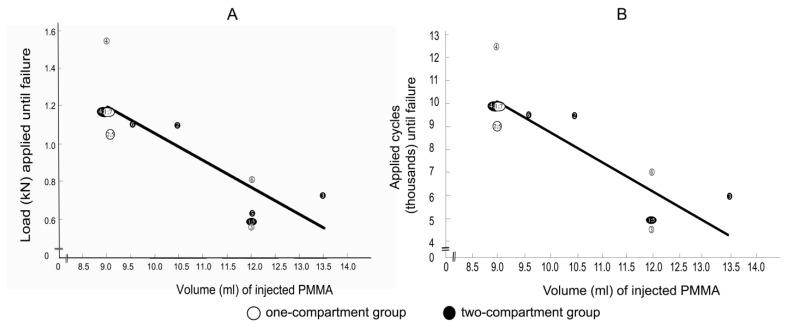
(**A**) correlations between applied load at failure and (**B**) between the number of applied cycles at failure with cement volume. Failure was defined by the radiological appearance of fractures adjacent to vertebra L1 (Figure 7) and/or by a compression depth of 7 cm underneath the starting position. Numbers refer to specimens. The Spearman correlation coefficients were −0.8 (*p* = 0.001) for both (**A**,**B**).

Spearman correlation coefficients between the applied load at the time of failure with the cement volume for the one-compartment group and for the two-compartment group were −0.805 (*p* = 0.029) and −0.806 (*p* = 0.053), respectively, and the correlation coefficients between the number of applied cycles at failure with the cement volume for the one-compartment group and for the two-compartment group were also −0.805 (*p* = 0.029) and −0.806 (*p* = 0.053), respectively. Considering that the correlation coefficients of the two groups were virtually identical, the values for the two groups were combined to increase statistical power and to shift the focus from the differences between the two groups to focus on the correlation between cement volume and failure. The Spearman correlation coefficients between the applied load at the time of failure and the cement volume (Figure 11A) and between the number of applied cycles until failure and the cement volume (Figure 11B) were both strongly negative and nearly linear.

## 4. Discussion

The goal of the new protocol was to simulate the early postoperative period. We applied up to 12,500 cycles, which would correspond to a week postoperative period in a young person and perhaps 10 to 14 days in an elderly person [1]. We were unaware of standardized protocols to evaluate the biomechanical properties of cadaveric spine samples subject to experimental loads. Some protocols used constant compression force [29,30], whereas others used increasing compression force [31,32], as did this study. We believe that the model reported here has several advantages compared with others. The biomechanical model that we developed combined increasing loading with cyclic loading to reduce testing time until failure. We envisaged that this approach could be used to facilitate further studies on spine fracture treatment of incomplete burst fractures.

Another difference in published protocols is the number of segments investigated. Multisegmented models of load-bearing capacity [33,34], such as the one used in this study, better simulate the actual spine than single-segmented models [29,35]. A multisegmented specimen can flex and extend. Plus, this study combined the advantage of using multisegmented specimens with increasing loading through cyclic loading until failure. In addition, this study’s compression protocol was based on radiographs taken in between each step. However, even though multisegmented specimens were used, the effects of changes in sagittal balance could not be addressed by our model.

The most important finding of this study was that the two-compartment device was not clearly superior to the one-compartment device.

### 4.1. Vertebral Height Reconstruction

In particular, we had expected to find greater vertebral height reconstruction in the two places that we measured in the posterior region of the vertebra, the posterior itself (Place a), and at one-third of the distance from the posterior of the vertebra to the anterior (Place b). Such a finding would have been interesting because Verlaan et al. found that increased disc survival was associated with anatomical endplate restoration [2]. In our study, kyphoplasty resulted in increasing height (endplate elevation) in all four measured places in vertebra L1. Therefore, vertebral height reconstruction by kyphoplasty was effective in both groups, the one-compartment group and the two-compartment group. However, increases in height were not statistically significantly different between the two groups at any of the four measured places. A possible explanation for not finding a significant difference in our study was that the study was not statistically powered to detect small differences. Availability of samples is limited in most biomechanical studies.

Despite the manufacturer’s instructions to use the same amount of water to fill the balloons in both the one- and two-compartment devices, the filling volume was significantly higher for the two-compartment device. The difference might have resulted from the surgeon’s effort to attain optimal reconstruction in view of the manufacturer’s statement that reconstruction of vertebral body height is more effectively accomplished using the two-compartment device.

Nonetheless, the extent of height reconstruction in our study was comparable with those of other studies using conventional kyphoplasty in cadaveric specimens and with clinical outcomes [15,29,36,37].

Holyoak et al. also failed to show any benefit for kyphoplasty with directional instruments considering the vertebral height restoration in comparison to conventional kyphoplasty [14].

Even a recently developed tool with the ability to directly manipulate the endplate did not show better reconstruction of the vertebral body height in comparison to conventional kyphoplasty, nor was any relevant reconstruction of the posterior wall found [12,13].

### 4.2. Vertebral Height Preservation

We had also hypothesized that kyphoplasty with the two-compartment device was superior to that with the one-compartment device as to the extent of load-bearing capacity until failure. Although the two-compartment device was inferior in this respect, the difference was not statistically significant.

Furthermore, although the two-compartment group had earlier adjacent fractures after cyclic loading than did the one-compartment group, the difference was not statistically significant. Therefore, we cannot conclude that the use of the one-compartment device would result in fewer fractures. However, the finding of lack of statistical significance might have resulted from a lack of significant power. The study of Holyoak et al. showed comparable results considering vertebral height preservation with no significant difference between conventional kyphoplasty and kyphoplasty with directional instruments, but a significant difference between kyphoplasty with directional instruments and augmented Kyphoplasty in favor of the first mentioned. However, there was also a correlating difference in the volume of cement used [14]. This is consistent with the findings of a recently published study by Sun et al. in which higher cement volume corresponds to better vertebral body height reconstruction [38]. Two other biomechanical studies of cadaveric vertebrae reported that height preservation depended on cement volume, in which a higher cement volume meant less height loss of the treated vertebra [39,40]. Thus, an increase in cement volume is possibly needed in an extensive height restoration to maintain the endplate.

### 4.3. Volume of Cement Correlates with Risk of Adjacent Fracture

Using a volume of cement appropriate for each kyphoplasty on a particular specimen rather than using a fixed volume, the surgeon was able to simulate an extent of height reconstruction similar to that observed in actual surgery. This was important because our studies on load-bearing capacity and cement volume were based on simulated clinical judgment. The volume of cement was higher for the two-compartment device, although not statistically significant. Compared with other experimental studies and with clinical data, the volume of cement was higher in this study, higher than the recommended volume of greater than 4.5 mL from Röder and colleagues [36,41,42,43]. However, the cement volumes reported in our study might have been slightly overestimated because of leakage resulting from lesions in the vertebra resulting from fracture.

Another key result we found is that increasing volume of cement was strongly correlated with an increasing risk of adjacent fractures after kyphoplasty.

There is controversy in the clinical literature regarding this result. Some clinical studies report that cement volume is not a risk factor for adjacent vertebral fractures after kyphoplasty. In a study by Ko et al., 123 patients underwent kyphoplasty, and 20 (16%) had early adjacent vertebral fractures (new fractures that had developed within 3 months after surgery). There was no difference in cement volume between these patients and those who did not have early adjacent vertebral fractures [44]. Teuber et al., and more recently, Essibayi et al., reported no difference in the risk of adjacent fractures after vertebroplasty or kyphoplasty compared to the natural cause of disease considering osteoporotic fractures [45,46]. Other clinical studies reported that higher cement volumes lead to a greater risk of unfavorable consequences after kyphoplasty. Yang et al. reported that larger cement volume is a risk factor for adjacent vertebral compression fracture after kyphoplasty [47]. A study by Yi et al. reported that two (2.5%) of the 79 kyphoplasty patients had subsequent adjacent fractures [48]. In these two patients, subsequent adjacent fractures occurred 20 days after kyphoplasty with 5 mL cement and at 2 months after kyphoplasty with 1 mL cement. Adida et al. were able to show a correlation between cement volume and adjacent level fracture in the thoracic spine but not in the lumbar spine [41]. Additionally, the use of less injected cement significantly boosted the potential risk of refracture [49]. A similar controversy exists considering the analgesic effect. Some studies report the use of higher cement volume to have a better analgesic effect, whereas others favor a reduced cement volume [38,43].

From a biomechanical perspective, the correlation between cement volume and adjacent fracture seems very plausible and in agreement with the previously reported literature data. Berlemann et al. reported that in vertebroplasty, the treated vertebra is fairly stiff, causing increased stress on the relatively soft adjacent osteoporotic vertebra [50]. This explanation also applies to kyphoplasty. Another study reported that strength and stiffness are weakly correlated with the percentage fill volume of cement injected [51]. A biomechanical study employing finite element analysis on an L1 vertebra reported that only a small amount of bone cement, about 15% fill volume, was necessary to restore the stiffness of the damaged vertebral body to the pre-damaged value [52]. The use of a 30% fill increased stiffness by more than 50% compared with the pre-damaged value. A greater filling can result in a substantial increase in stiffness well beyond the pre-damaged value and might not be the most biomechanically optimal. Another study using finite element analysis described that the cement distribution within the vertebral body corresponds to stress on the adjacent segment [53]. Macciachera reported in a review that mechanical percutaneous vertebral body augmentation (MPVA) reduced the rates of adjacent fractures compared to conventional kyphoplasty [54]. This is consistent with a difference in stiffness between solid cement and an MPVA. Therefore, future studies should report the biomechanical parameters of the cement used, and patient-specific adjustment of the kyphoplasty cement could reduce the risk of an adjacent fracture [55]. Currently, the intravertebral devices for reconstruction of the vertebral endplate reported in literature and this study do not significantly influence vertebral height preservation and risk of adjacent fracture.

### 4.4. Limitations

Biomechanical studies are based on a model and do not necessarily reflect clinical conditions. Vertebral reconstruction by kyphoplasty in this study is undertaken with upright specimens, whereas clinically, the patient is in the prone position. As mentioned, we attempted to account for differences in pressure.

The protocol in this study was intended to simulate physiologic conditions during the early postoperative period after kyphoplasty to determine the accumulated load until failure.

To develop our model simulating the accumulated load until failure, we considered previously reported observations. Wilke et al. applied 100,000 cycles of constant load in one loading test to cadaveric specimens to measure the loss of height [1]. They stated that applying such a load simulated a 5-to 6-week postoperative period in a young patient and a 3-month period in an elderly patient. Decomposition of thawed postmortem specimens limits the amount of time to apply cyclic loading until failure. This is a limitation of studies on postmortem specimens, and we were not aware of a specific time limitation. Therefore, in our study, continually increasing loading until failure was applied in one cyclic loading test. Otherwise, either failure might not have occurred, or testing until failure might have taken too long. Therefore, as with previous work, the time until failure in our work was not comparable with a patient’s experience [1].

Another important factor of cyclic testing is the frequency of cyclic loading. Hansson et al. used a frequency of 0.5 Hz to simulate physiologic conditions [56]. However, there was no standard in the frequency used in previous studies, which used frequencies ranging from 0.25 to 5 Hz [29,30,31,32,57]. We used an in-between value of 2 Hz because we were unaware of validated recommendations. The values of increasing compressive load used in this study were based on published values during standing (600 N) and normal walking (900 N) [23] up to maximal loads until vertebral fracture, 1040 N based on calculation [58] and about 2500 to 3500 based on measurement of fracture load [32].

We developed a method to simulate repetitive loads to a multisegmental specimen in an effort to attempt to assure that load until failure was reached within the time range of usage from freshly frozen cadaveric specimens. Previous studies used either cyclic loading [1,29,30,31,42,56,57] or steady compressive force [7,36] to simulate repetitive loads onto a multisegmental specimen and to ensure that load until failure was reached within the time range of usage from freshly frozen cadaveric specimens. Our method combined these factors. Inasmuch as decomposition is an ongoing process, we sought to measure endpoint values as soon as possible to simulate physiological conditions as much as possible. However, to our knowledge, a time range of limitations on making such observations has not been reported. All specimens were tested under the same conditions, so we believe that it is reasonable to compare findings from one specimen to another. However, applying these findings to patients would not be accurate. This is a limitation of biomechanical studies of postmortem specimens.

## 5. Conclusions

In accordance with the intravertebral devices reported in the literature, no advantage for the two-compartment device with regard to reconstruction and preservation of the vertebral body height and the vertebral endplate could be demonstrated in the present study. Due to high stiffness, the cement volume and not the intravertebral device seem to have the decisive influence on vertebral height preservation and the risk of adjacent fractures, creating a clinical dilemma in spine surgery that has yet to be resolved.

## Figures and Tables

**Table 1:** Decreases in height (mm) of vertebra L1 after compression.

Place * in Vertebra L1		a		b		c		d	
		Height (mm)		Height (mm)		Height (mm)		Height (mm)	
		Before	After	Before	After	Before	After	Before	After
Group	Specimen	compression		compression		compression		compression	
one compartment	1	23.84	24.47 †	24.87	19.31	24.36	17.63	20.68	21.25 †
	2	26.91	26.49	24.85	22.28	24.26	20.57	22.07	19.99
	3	28.12	27.22	27.21	21.86	26.52	20.95	24.26	18.70
	4	28.32	26.03	25.62	22.92	25.31	22.43	27.52	27.02
	5	28.84	27.12	22.97	19.86	21.80	18.31	22.47	18.80
	6	28.97	23.88	27.41	20.01	26.28	18.47	27.02	18.26
	7	29.10	27.06	27.22	21.17	27.23	20.90	26.80	19.86
two compartment	1	27.91	23.24	24.19	18.52	24.82	18.56	26.11	19.06
	2	28.31	24.87	27.44	22.11	26.98	21.97	25.77	21.94
	3	28.37	24.83	27.09	20.60	26.26	19.13	27.77	16.94
	4	28.63	26.59	26.50	22.37	25.72	21.45	25.10	19.81
	5	28.84	26.07	26.95	19.03	26.26	17.29	26.44	16.48
	6	30.29	28.34	26.93	22.00	25.86	19.92	28.42	19.64
Comparison between before and after compression by using Mann–Whitney U-Test		*p* = 0.86		*p* = 0.32		*p* = 0.39		*p* = 0.86	

* Heights were measured at the four places shown in Figure 6. † As noted in the text, the increases in length after compression at these two places were caused by measuring error.

**Table 2:** Increases in height (mm) of vertebra L1 from after compression to after kyphoplasty.

Place * in Vertebra L1		a		b		c		d	
		Height (mm)		Height (mm)		Height (mm)		Height (mm)	
		Before	After	Before	After	Before	After	Before	After
Group	Specimen	kyphoplasty		kyphoplasty		kyphoplasty		kyphoplasty	
One compartment	1	24.47	24.76	19.31	21.88	17.63	20.87	21.25	21.91
	2	26.49	27.36	22.28	23.49	20.57	23.45	19.99	21.99
	3	27.22	27.12 ‡	21.86	22.30	20.95	21.91	18.70	20.39
	4	26.03	27.22	22.92	23.25	22.43	23.19	27.02	26.81 ‡
	5	27.12	28.64	19.86	23.14	18.31	20.97	18.80	22.47
	6	23.88	24.75	20.01	20.49	18.47	19.71	18.26	23.53
	7	27.06	27.54	21.17	23.37	20.90	23.50	19.86	26.02
Two compartment	1	23.24	25.26	18.52	20.71	18.56	21.11	19.06	22.45
	2	24.87	27.58	22.11	24.32	21.97	24.44	21.94	23.24
	3	24.83	26.80	20.60	23.17	19.13	23.20	16.94	22.59
	4	26.59	27.19	22.37	23.49	21.45	22.78	19.81	20.96
	5	26.07	26.00 ‡	19.03	21.18	17.29	19.72	16.48	19.21
	6	28.34	28.86	22.00	25.13	19.92	23.83	19.64	25.70
Comparison between before and after compression by using Mann–Whitney U-Test		*p* = 0.317		*p* = 0.317		*p* = 0.317		*p* = 0.568	

* Heights were measured at the four places shown in Figure 6. ‡ As noted in the text, the decreases in length after kyphoplasty at these three places were caused by measuring error.

## Data Availability

Dataset available on request from the authors.
